# Prediction of Osteoporosis at the Sacrum Using Opportunistic CT of the Abdomen and Pelvis: A Retrospective Feasibility Study in 277 Patients Comparing CT and QCT Data

**DOI:** 10.3390/jcm15093473

**Published:** 2026-05-01

**Authors:** Yan Xiao, Wen Li, Wenqin Zhou, Miao Wei, Bangyuan Long, Jiayi Pu, Fajin Lv

**Affiliations:** 1 Department of Radiology, Bishan Hospital of Chongqing Medical University, Chongqing 420760, China; 2 Medical Imaging Department, Yubei District Traditional Chinese Medicine Hospital, Chongqing 401120, China; 3 Department of Radiology, The First Affiliated Hospital, Chongqing Medical University, Chongqing 400016, China; 4 Department of Radiology, Chongqing General Hospital, Chongqing University, Chongqing 400013, China

**Keywords:** computed tomography, QCT, BMD, osteoporosis, sacrum, Hounsfield unit (HU)

## Abstract

**Summary** This study assessed the use of opportunistic abdominopelvic computed tomography (CT) for the evaluation of the sacrum as a predictive tool for osteoporosis. Sacral spine Hounsfield unit (HU) values measured by CT showed good correlation with mean bone mineral density (BMD) for L1–L2 measured by quantitative computed tomography (QCT), and good diagnostic performance for the identification of osteoporosis. The results of this study suggest that it is possible to obtain comprehensive information on bone health in individuals who undergo CT of pelvic. **Objectives** To examine the distribution pattern of bone density in the L1–S3 vertebrae using opportunistic abdominopelvic imaging. QCT was employed as a reference to establish HU thresholds for the sacral vertebrae facilitating the prediction of osteoporosis and the exclusion of bone abnormalities. **Methods** A total of 277 subjects aged 19 to 81 years who underwent abdominopelvic CT were evaluated. Bone mineral density (BMD) measurements for the L1–S3 vertebrae and HU values for the S1–S3 vertebrae were collected. The study analyzed the correlation between sacral spine HU values and sacral spine BMD, along with the clinically utilized mean BMD for L1–L2, was analyzed. Receiver operating characteristic (ROC) curves were generated to identify the optimal diagnostic thresholds. **Results** The BMD of the lumbosacral vertebrae displayed a gradual decrease from L1 to L3, followed by an increase from L4 to S1, and a subsequent decline from S1 to S3. HU values of the sacral vertebrae across all planes were strongly correlated with both sacral spine BMD and the mean BMD values for L1–L2 (r = 0.830 to 0.905, *p* < 0.05). For individual vertebrae, the area under the curve (AUC) of HU values for predicting osteoporosis ranged from 0.909 to 0.977, while the AUC for excluding bone abnormalities ranged from 0.933 to 0.950, with S1 demonstrating the highest predictive efficacy. The optimal threshold for S1 was >165.17 HU, yielding a specificity of 91.5% and a sensitivity of 83.0% for excluding bone abnormalities. Conversely, an S1 threshold of <130.50 HU resulted in a diagnostic specificity of 90.0% and a sensitivity of 96.6% for osteoporosis. Additionally, a predictive model that incorporated sex, age, and vertebral cancellous bone HU values achieved an AUC of 0.981. **Conclusions** Our data demonstrate a strong correlation between the HU values of the sacral spine and the clinically used BMD values for L1–L2, supporting the prediction of osteoporosis based on sacral spine HU values. Moreover, a predictive model that includes sex, age, and vertebral measurements offers improved diagnostic accuracy.

## 1. Introduction

Osteoporosis is a systemic metabolic bone disease characterized by reduced bone mass and microstructural deterioration, leading to increased bone fragility. This condition has a significant impact on patients’ quality of life and can pose direct and indirect risks to life [1,2]. The prevalence of osteoporosis is rising rapidly due to an aging population and lifestyle changes, evolving into a global health concern that now affects over 200 million people [3]. Early screening for osteoporosis is essential for timely diagnosis and treatment, which can help lower the risk of fractures among high-risk individuals. However, current osteoporosis diagnosis rates remain low, with many cases only being detected at advanced stages, when fractures have already developed [4].

Dual-energy X-ray absorptiometry (DXA) is recommended by the World Health Organization (WHO) as the gold standard for the diagnosis of osteoporosis [5]. However, DXA results may be influenced by factors such as osteomalacia and vascular calcification, and a growing body of research has highlighted the limitations of relying exclusively on DXA for osteoporosis screening and fracture risk assessment [6,7]. Quantitative computed tomography (QCT) offers a widely used method of bone densitometry, enabling separate measurements of bone density in cortical and cancellous bone, which provides greater accuracy than DXA [8]. Despite these benefits, QCT requires additional scans and results in a higher radiation dose. With the extensive use of computed tomography (CT) and rising healthcare costs, the application of conventional CT imaging for opportunistic screening in at-risk populations has gained broad acceptance [9,10,11,12]. By measuring the Hounsfield units (HU) of vertebrae, bone density can be indirectly estimated, which vary considerably across spinal regions (cervicothoracic-lumbar spine). Recently, dual-energy CT (DECT) provides more accurate BMD quantification and osteoporosis diagnosis by material decomposition and reducing HU biases, its limited scanner availability and need for specialized post-processing hinder large-scale clinical application, making widespread single-energy CT still practically valuable for opportunistic screening [13,14].

Opportunistic screening for osteoporosis using routine chest and abdominal computed tomography (CT) has been well established and validated in previous research [10,11,12]. Given the analogous anatomical characteristics of the sacrum relative to the lumbar spine, as well as the extensive clinical utilization of lower abdominal and pelvic CT examinations, measuring Hounsfield unit (HU) values derived from pelvic CT scans represents a promising approach for evaluating osteoporosis. In clinical practice, pelvic CT is frequently obtained for the assessment of low back pain—symptoms that may in fact be secondary to underlying osteoporosis. Nevertheless, such imaging studies are typically interpreted with a primary focus on abdominopelvic organ pathologies, rather than the structural and mineral density status of the lumbosacral spine. Consequently, the implementation of pelvic CT-based bone mineral density assessment holds substantial clinical value, and may enable large-scale opportunistic osteoporosis screening in routine clinical care.

As a result, our objectives were as follows: (1) to explore the bone density distribution of the L1–S3 segment via opportunistic abdominopelvic CT; (2) to determine sacral HU thresholds for osteoporosis screening and the exclusion of bone abnormalities.

## 2. Methods

### 2.1. Patients

This study retrospectively analyzed patients undergoing CT scans at the Department of Radiology at the First Hospital of Chongqing Medical University from September 2022 to September 2025. The inclusion criteria were: (1) age ≥ 18 years, and (2) completion of at least one CT examination encompassing the lumbosacral region. Exclusion criteria included: (1) presence of spinal tuberculosis, bone tumors, ankylosing spondylitis, dense osteitis, or diffuse idiopathic osteomalacia; (2) history of lumbosacral vertebral fractures or surgeries; (3) other conditions affecting bone metabolism; and (4) anatomical variations of the lumbosacral vertebrae. This retrospective study was approved by the institutional review board. Written informed consent was not required from patients.

### 2.2. CT Protocol and HU Values Measurements

**Figure 1:**
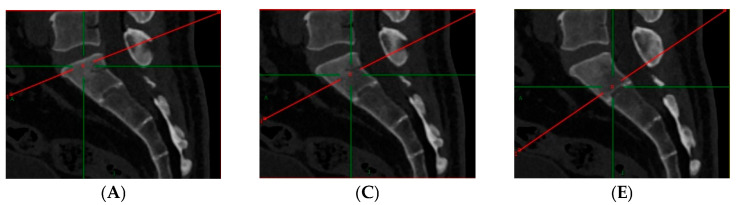

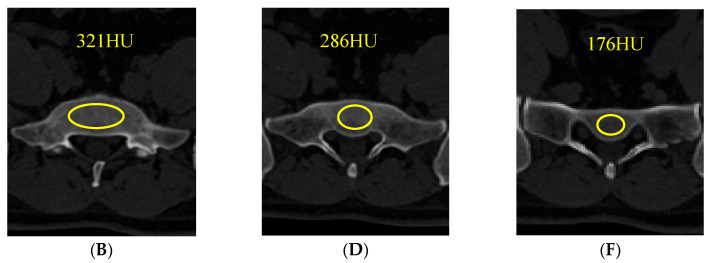
Measurement of Hounsfield unit description: (**A**,**B**) axial section of the cranial region (HU); (**C**,**D**) axial section of the middle region (HU); (**E**,**F**) axial section of the caudal region (HU). Yellow circles: region of interest (ROI) and corresponding parameters (HU value). Red/Green lines: sagittal/coronal/axial localization lines.

All CT examinations were conducted using a Siemens SOMATOM Force CT scanner. The scanning parameters were set as follows: tube voltage at 120 kV, automatic tube current, slice thickness of 5 mm, and reconstruction of 1 mm thin-slice images. To maintain the accuracy of measurements, the scanners were regularly calibrated using standard musculoskeletal module protocols. Picture Archiving and Communication System (PACS, Siemens) was used for CT measurements, using both axial and sagittal imaging positions. The measurement range included the L1–S3 vertebrae, and the CT measurement methodology involved manually outlining the region of interest (ROI) in the axial position, with subsequent adjustments in the sagittal position. The ROIs were selected at three specific levels of the vertebral body: just below the upper endplate, at the midpoint of the vertebral body, and just above the lower endplate. Efforts were made to exclude the bone cortex, isolated bone structures, hardened regions due to osteochondrosis, and the posterior central venous groove of the vertebral body. The ROI was defined to encompass the cancellous bone region as thoroughly as possible (Figure 1). For each vertebra, HU values were measured at the three axial levels, and an average HU value was then calculated and recorded in Hounsfield units (HU). The individual conducting the measurements was blinded to the subjects’ bone densitometry results to prevent any potential subjective bias in data collection. All measurements were performed twice by the same observer to assess intra-observer reliability.

### 2.3. BMD Measurement

**Figure 2:**
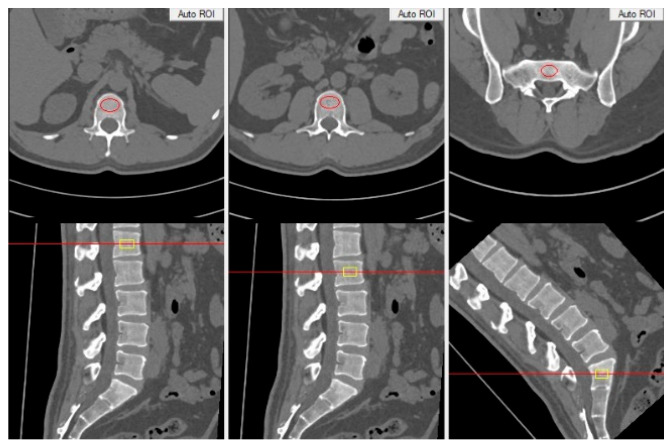
Measurement of bone mineral density (BMD).

QCT measurements were conducted using the American Mindways (MW) QCT PRO V6.1 bone density analysis software. The 1-mm thin-slice CT images of the lumbosacral vertebrae were imported into the MW software. Using multiplanar reformation, axial images of the vertebral body at the central level were selected with reference to both coronal and sagittal planes. Regions of interest (ROIs) were manually outlined to exclude bone cortex, isolated bone structures, hardened areas such as osteophytes, and the posterior central venous grooves, ensuring the ROIs covered as much cancellous bone as possible (Figure 2). The MW software then measured the BMD of vertebral cancellous bone from L1 to S3, automatically generating a BMD value for each vertebra in mg/cm^3^. Based on the diagnostic criteria for osteoporosis using lumbar spine QCT [15], patients were categorized into three groups on the basis of their BMD values: osteoporosis (BMD ≤ 80 mg/cm^3^), osteopenia (80 mg/cm^3^ < BMD < 120 mg/cm^3^), and normal (BMD ≥ 120 mg/cm^3^).

### 2.4. Statistical Methods

The statistical analysis was performed using SPSS version 29.0 software(IBM, Armonk, NY, USA). Data normality was evaluated with the Shapiro-Wilk (S-W) test, which indicated that some of the sacral spine BMD and HU values did not follow a normal distribution; these values were thus reported as medians and interquartile ranges [M (P25, P75)]. Interobserver measurement agreement was assessed using linear regression analysis and Bland-Altman analysis. Spearman’s correlation coefficient was used to analyze the relationship between the HU values of the sacral vertebrae, sacral spine BMD, and the mean BMD of L1–L2. Logistic regression analysis was conducted to develop a model incorporating multiple indicators for predicting osteoporosis and excluding bone abnormalities. Optimal thresholds for excluding bone abnormalities and diagnosing osteoporosis were identified for each sacral vertebra using receiver operating characteristic (ROC) curves. The predictive value of sacral vertebrae HU values for normal and osteoporosis was assessed based on the area under the curve (AUC), with a *p*-value of less than 0.05 considered statistically significant.

## 3. Results

### 3.1. Differences in BMD and HU Values of the Lumbosacral Spine Among Patients with Normal, Osteopenia, and Osteoporosis

After applying the inclusion and exclusion criteria, 277 patients in all, aged 19 to 81 years, with a mean age of 51.98 ± 12.21 years, were included in the study. This cohort consisted of 235 females and 42 males. Based on QCT diagnostic criteria, participants were classified into three groups: normal (141 cases), osteopenia (83 cases), and osteoporosis (53 cases).

**Figure 3:**
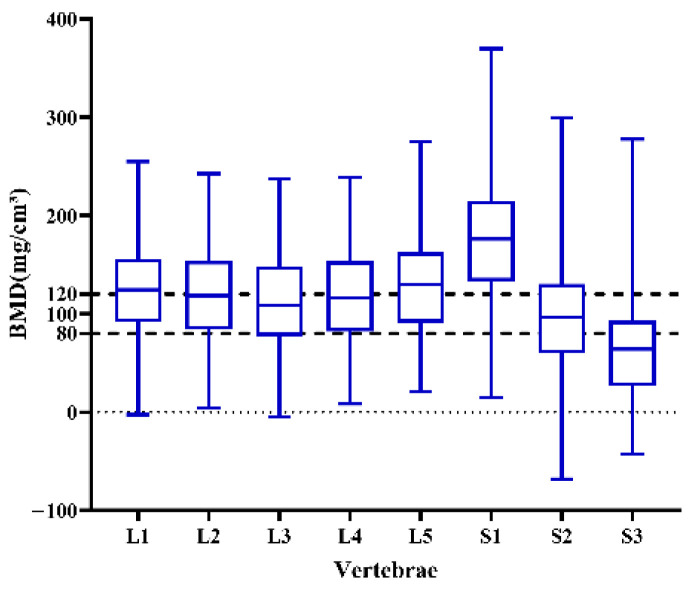
Trend distribution of BMD in the lumbosacral spine.

**Figure 4:**
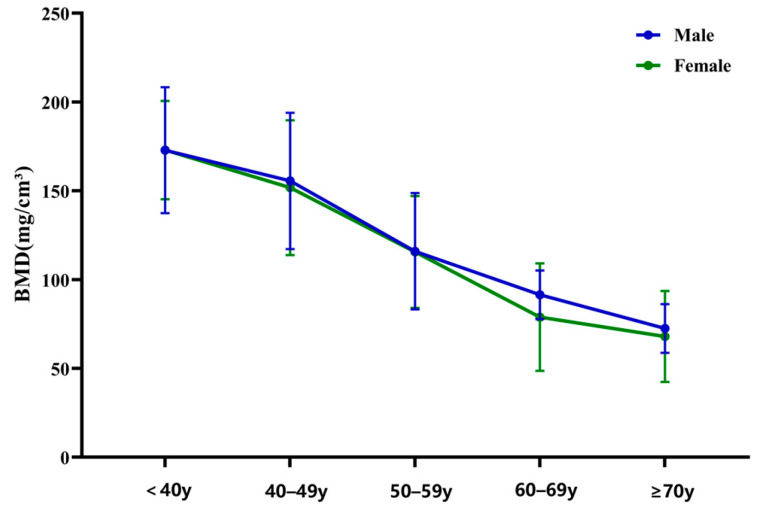
Comparison of BMD values for males and females in different age groups.

The bone density of the lumbosacral vertebrae showed a gradual decline from L1 to L3, an increase from L4 to S1, and a subsequent decrease from S1 to S3. The highest bone density was observed in the S1 vertebra at 176.43 (132.77, 214.91) mg/cm^3^, as illustrated in (Figure 3). BMD values of the lumbosacral vertebrae decreased with age. Although males generally exhibited higher BMD than females across various age groups, particularly after age 50, the differences were not statistically significant (*p* > 0.05) (Figure 4).

**Figure 5:**
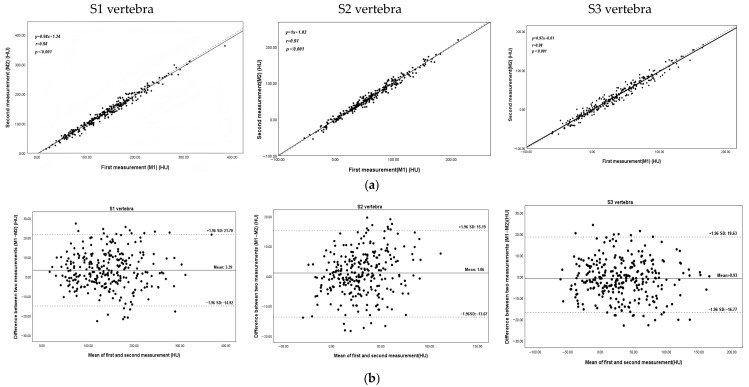
Inter-observer agreement between the two measurements and Bland-Altman plots of S1–S3 vertebrae. (**a**) Scatter plot showing the correlation between two measurements across S1–S3 vertebrae. Each point represents the mean HU value for one patient. The solid line indicates the line of identity (y = x), and the dashed line represents the linear regression fit. (**b**) Bland-Altman plot demonstrating the agreement between first measurement (M1) and second measurement (M2). The solid horizontal line indicates the mean difference (M1–M2), and the dashed lines indicate the upper and lower 95% limits of agreement. All HU values are expressed in HU.

Intra-observer agreement between the two measurements was excellent, with correlation coefficients (r) ranging from 0.97 to 0.98 (Figure 5). Table 1 shows the HU values of the sacral spine across the subgroups defined by QCT as normal, osteopenia, and osteoporosis. A statistically significant difference (*p* < 0.05) was observed in the cancellous bone HU values of the sacral spine among the three groups, with the HU values of the S1–S3 vertebral bodies exceeding those of the corresponding BMD values. In addition, the percentage of females gradually increased in the three groups, and the average age also rose progressively within each group.

### 3.2. Correlation Analysis Between HU Values of the Sacral Vertebrae and Age, as Well as the BMD of the Sacral Vertebrae and the Mean BMD Values of L1–L2

The HU values of each sacral vertebra demonstrated a significant negative correlation with age (*p* < 0.001) (Table 2), consistent with the established inverse relationship between BMD and age. Additionally, a strong positive correlation was observed between the HU values of the sacral vertebrae and both the BMD values of the sacral vertebrae and the mean BMD values of L1–L2. The highest correlation was found between the HU values of S1 and the mean BMD values of L1–L2 (r = 0.905, *p* < 0.01), while the weakest correlation was noted between the HU values of S3 and the mean BMD values of L1–L2 (r = 0.844, *p* < 0.01). Notably, the correlation between the HU values of S2 and BMD was particularly strong (r = 0.900, *p* < 0.01).

### 3.3. Prediction of Osteoporosis and Exclusion of Bone Abnormalities Through Logistic Regression Analysis of HU Values in Sacral Vertebrae

**Figure 6:**
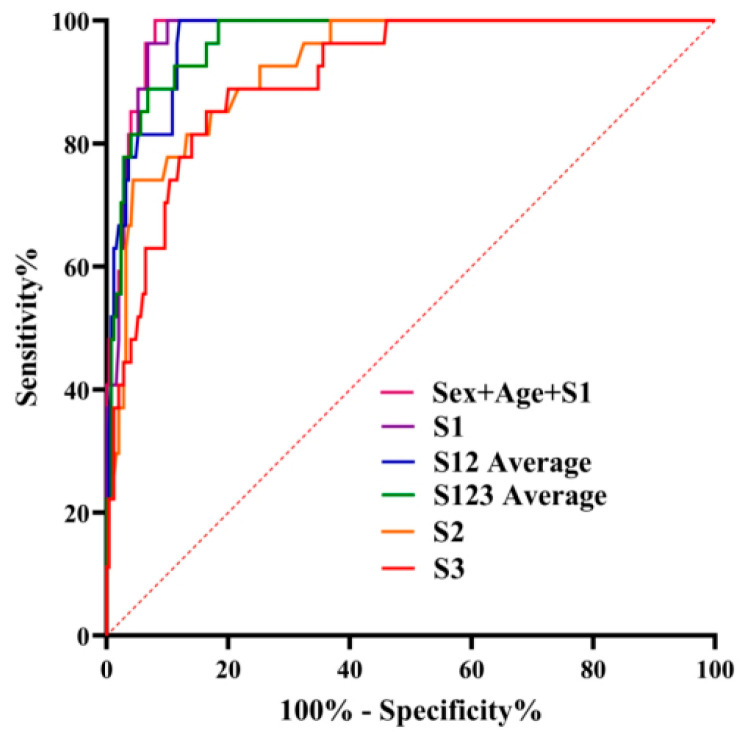
Receiver operating curve (ROC) of HU values for prediction of osteoporosis risk.

For individual vertebrae, HU values exhibited excellent diagnostic performance in predicting osteoporosis, with AUC values ranging from 0.909 to 0.977. Meanwhile, the AUC for excluding bone abnormalities ranged from 0.933 and 0.950 (Table 3). The S1 vertebra displayed the highest AUC for both predicting osteoporosis and excluding bone abnormalities, exceeding the values observed for the S2 and S3 vertebrae. The optimal HU threshold for diagnosing osteoporosis at the S1 level was determined to be 130.50 HU, with a specificity of 90% and a sensitivity of 96.6%. In contrast, the optimal threshold for excluding bone abnormalities was identified as 165.17 HU, yielding a specificity of 91.5% and a sensitivity of 83.0%. Furthermore, when incorporating gender, age, and the S1–S3 vertebrae into the regression model for osteoporosis prediction, the combined model including age, gender, and the S1 vertebra produced the highest AUC value of 0.981, as illustrated in Figure 6.

## 4. Discussion

While both QCT and DXA are valuable tools for measuring BMD and predicting osteoporosis, each has its limitations. Opportunistic CT, on the other hand, utilizes existing clinical imaging data to assess bone density and screen for osteoporosis, without incurring additional costs or radiation exposure for patients, thus supporting its broader use in clinical practice [16,17,18]. Routine abdominopelvic CT scans, commonly employed for diagnosing and managing various diseases, generate images that offer meaningful insights into vertebral quality. This study evaluated the use of sacral vertebral HU values from abdominopelvic CT to predict BMD and established a threshold HU value for diagnosing osteoporosis in the sacral vertebrae. Our findings demonstrated a strong correlation between sacral spine HU values measured through opportunistic CT and the clinically accepted diagnostic criterion of mean BMD for L1–L2. Moreover, sacral spine HU values proved to be an effective metric for identifying patients with osteoporosis.

In this study, BMD was typically lower for females than for males within the same age group, with significant differences becoming apparent after age 50. The proportion of females increased progressively across the groups from normal to osteopenia and, ultimately, to osteoporosis. This observation aligns with the well-known susceptibility of women over 50 to osteoporosis [19], which is often attributed to the postmenopausal decline in estrogen levels that negatively impacts bone mineral density in females [20]. Additionally, Jang et al. [14] reported a negative correlation between HU values and age. Consistent with these findings, our study showed that as age advances, bone mass reduces, trabecular bone thins, bone density declines, and the HU values of the vertebrae decrease accordingly.

The findings of our study confirm significant variability in bone density across the lumbosacral vertebrae. Specifically, we observed a gradual decline in bone density from L1 to L3, followed by an increase from L4 to S1, which aligns closely with previous research [12]. Additionally, our study demonstrated a progressive decrease in bone density from S1 to S3 within the sacral vertebrae, with the S1 vertebra displaying significantly higher density than the other lumbosacral vertebrae. This difference may be due to the substantial weight-bearing load supported by the S1 vertebra. Previous studies have established a strong correlation between lumbar vertebral BMD and that of the cervicothoracic vertebrae [21], suggesting a similar correlation between the sacral and lumbar vertebrae. This hypothesis was supported by our results, with correlation coefficients ranging from r = 0.844 to 0.90 (*p* < 0.05). In our analysis, patients were classified into three groups based on BMD measured by QCT: normal, osteopenia, and osteoporosis. Statistically significant differences were detected in the sacral vertebral HU values among these groups, a result that contrasts with findings reported by Ping Wang [22]. We speculate that these differences may be due to variations in study populations and equipment used.

Vertebral HU values are not only a useful supplementary tool for diagnosing osteoporosis [23,24,25] but also for assessing high fracture risk [26]. Established diagnostic thresholds for osteoporosis in the thoracic spine are 133.01 HU, with 208.85 HU as the threshold for excluding abnormal bone mass [24]. In the lumbar spine, the threshold for diagnosing osteoporosis is 106.38 HU, with a diagnostic specificity of 92.6% [27]. A prior study that evaluated the diagnostic effectiveness of the L4-S1 vertebrae for osteoporosis using abdominal CT and DXA in 50 patients reported an AUC of 0.65 for the S1 vertebra in predicting osteoporosis, with thresholds set at 207 HU for normal and 68 HU for osteoporosis [28]. In contrast, the present study identified 165.17 HU and 130.50 HU as thresholds for S1 to exclude bone abnormalities and diagnose osteoporosis, respectively, differing notably from prior findings. These threshold differences may stem from variations in race, equipment, measurement techniques, and sample sizes across studies. In this study, we combined age, sex, and sacral vertebrae HU values to predict osteoporosis, finding that this multi-factor model provided greater predictive accuracy than using single-segment vertebrae alone (combined AUC = 0.981 vs. single vertebrae AUC = 0.909–0.977). However, the absence of external validation and potential confounding factors such as age and sex in the study may have led to an overestimation of the results. Additionally, this study compared the predictive capabilities of single-segment sacral vertebrae with multi-vertebrae combinations for osteoporosis, showing that single-segment predictions outperformed those of multi-vertebrae unions. We hypothesize that the smaller size of certain sacral vertebrae may hinder complete elimination of vertebral cortical bone influence during manual measurement. Considering that vertebral HU values can differ between manual and fully automated methods [29], further studies using software for precise segmentation of cortical and cancellous bone in the sacral vertebrae are recommended.

Osteoporotic fractures predominantly impact the thoracic and lumbar spine, which has led to a clinical focus on thoracolumbar BMD. However, the sacral spine can be especially susceptible to bone loss due to its relatively low mobility compared to other vertebrae and the lack of mechanical loading needed for bone remodeling and repair. Osteoporosis in the sacral spine often manifests as asymptomatic lower back pain, which can easily be misinterpreted as pain from lumbar disc degeneration or genitourinary disorders [30]. Thus, assessing bone density in the sacral spine is highly significant. Like the lumbar spine, the sacral spine undergoes degenerative changes. This study demonstrates that while absolute values may vary, the pattern of bone density reduction over time is comparable across different spinal regions. Rapid and accurate identification of abnormal bone density before sacral surgery is crucial, as recent studies indicate that postoperative screw loosening in the spine is linked to low BMD, highlighting the importance of BMD assessment in the sacral spine [31].

This study has several limitations: (i) It is a retrospective, single-center and single-device study with a small sample size, potentially introducing selection bias and limiting the generalizability of our results. In the future, multi-center and multi-device investigations involving large sample sizes will be conducted. (ii) Our study focused on the predictive efficacy of specific sacral spine HU values for diagnosing osteoporosis, without examining the performance of these HU values in predicting fractures. Future studies will aim to address fracture risk among our subjects. (iii) Current vertebral BMD assessments are primarily conducted in the lumbar spine, and measuring HU values in the sacral spine serves only as an opportunistic approach for high-risk individuals. (iv) Using QCT instead of DXA might affect the comparison with clinical diagnostic standards.

## 5. Conclusions

In conclusion, given the strong correlation between sacral spine HU values and the clinically utilized mean BMD values of L1–L2, osteoporosis can be diagnosed based on sacral spine HU values. Additionally, the combined prediction model that includes age, sex, and vertebrae provides greater accuracy than assessments based on individual vertebrae alone. Therefore, we believe that measuring sacral HU values on routine pelvic CT scans may serve as a promising screening tool for osteoporosis. This approach can assist clinicians in selecting at-risk patients for more precise BMD examinations, supporting early osteoporosis diagnosis and the prevention of fragility fractures.

## Figures and Tables

**Table 1:** The HU values and BMD values of Sacral Spine based on QCT bone density classification.

Projects	Classification Based on QCT Bone Density (277)		
	Normal (141)	Osteopenia (83)	Osteoporosis (53)
Sex (n)			
Male	24 (17%)	12 (14%)	6 (11%)
Female	117 (83%)	71 (86%)	47 (89%)
Age/year	46.00 (37.00,52.00)	57.00 (52.00,61.000)	64.00 (58.00,68.00)
S1-HU values/HU	246.00 (219.67,276.67)	168.67 (151.33,189.67)	120.33 (101.33,142.67)
S2-HU values/HU	129.41 (108.40,59.51)	74.60 (56.25,94.27)	41.68 (30.79,54.40)
S3-HU values/HU	104.00 (82.76,136.17)	44.67 (27.33,62.00)	20.33 (−8.50,35.34)
S1-BMD values/(mg/cm^3^)	212.10 (187.62,234.11)	151.63 (130.30,174.17)	100.38 (80.20,127.86)
S2-BMD values/(mg/cm^3^)	128.67 (108.17,162.84)	65.00 (48.67,82.33)	25.67 (14.67,50.00)
S3-BMD values/(mg/cm^3^)	90.54 (65.77,119.49)	43.02 (19.44,68.16)	14.74 (−2.15,32.37)
HU average values/HU	158.44 (142.00,190.39)	93.56 (78.56,109.44)	58.67 (41.83,71.67)
BMD average values/(mg/cm^3^)	143.38 (96.92,199.42)	84.43 (49.83,130.71)	45.51 (19.03,88.29)

Note: HU-Hounsfield units, BMD-bone mineral density, HU average-mean HU values based on CT measurements for S1–S3 vertebrae, BMD average-mean BMD values based on QCT measurements for S1–S3 vertebrae.

**Table 2:** Correlation between HU values of sacral spine with age, sacral spine BMD values and mean L1–L2 BMD values.

HU Values	Age	Sacral Spine BMD Values	L1–L2 BMD Average Values
S1	−0.742 **	0.890 **	0.905 **
S2	−0.670 **	0.900 **	0.881 **
S3	−0.666 **	0.830 **	0.844 **

Note: ** *p* < 0.001.

**Table 3:** ROC analysis to identify patients with or without osteoporosis and to detect the presence of bone abnormalities.

Classifications	Mark	Cut-Off Value	*AUC*	95%*CI*		*p*	Specificity	Sensitivity	Jordon Index
				Lower Limit	Up Limit				
Osteoporosis									
	Sex + Age + S1	-	0.981	0.967	0.994	<0.001	0.920	0.988	0.912
	S1	130.50	0.977	0.961	0.992	<0.001	0.900	0.966	0.889
	S12	96.00	0.970	0.950	0.990	<0.001	0.880	0.973	0.868
	S123	65.17	0.968	0.946	0.990	<0.001	0.932	0.889	0.821
	S2	36.17	0.928	0.886	0.970	<0.001	0.956	0.741	0.697
	S3	26.00	0.909	0.860	0.958	<0.001	0.800	0.889	0.689
bone abnormalities									
	S1	165.17	0.950	0.926	0.973	<0.001	0.915	0.830	0.745
	S2	84.84	0.947	0.923	0.970	<0.001	0.831	0.920	0.751
	S3	59.165	0.933	0.906	0.960	<0.001	0.810	0.955	0.765

## Data Availability

The original contributions presented in this study are included in the article. Further inquiries can be directed to the corresponding author(s).
